# Improving renal transplant outcomes through the tubeless anesthesia technique in renal transplantation: a retrospective study

**DOI:** 10.1186/s12871-025-03441-z

**Published:** 2025-11-20

**Authors:** Chuanbao Chen, Jingfeng Ou, Guanyi Wu, Hui Liu, Hanyu Yang, Shaojie Fu, Zhihong Ran, Junbiao Xie, Xin Xu, Jianxing He, Chao Yang, Xiaoyou Liu

**Affiliations:** 1 Department of Organ Transplantation, The First Affiliated Hospital, Guangzhou Medical University, Guangzhou, China; 2 Department of Anesthesiology, The First Affiliated Hospital, Guangzhou Medical University, Guangzhou, China

**Keywords:** Tubeless surgery, Tubeless anesthesia, Kidney transplantation, Clinical outcome

## Abstract

**Objective:**

To evaluate the feasibility and benefits of tubeless anesthesia (TA), using a laryngeal mask airway instead of endotracheal intubation (ETT), in renal transplantation, aligning with tubeless surgery principles.

**Methods:**

A single-center, retrospective matched-cohort study compared perioperative outcomes and 90-day graft function between TA and ETT in kidney transplantation. 52 adult recipients (26 TA, 26 ETT) operated between July-December 2024 were included. Groups were balanced for age, BMI, ASA classification, and dialysis duration. The Mann-Whitney U test and T test (SPSS 22.0) were used to analyze the outcome indicators: intraoperative anesthetic management, hemodynamics, recovery parameters, and graft function.

**Results:**

The TA group required significantly lower median doses of cisatracurium (12.6 mg vs. 26.1 mg; *p* < 0.001) and sufentanil (22.3 µg vs. 28.7 µg; *p* = 0.026). Operative times were similar (211.5 min vs. 200.8 min; *p* = 0.475). Vasoactive agent use was lower in the TA group (53.8% vs. 73.1%; *p* = 0.211), with fewer requiring dual agents (3.8% vs. 15.4%). TA patients exhibited faster awakening (recovery time: 18.5 min vs. 34.4 min; *p* < 0.001) and fewer airway complications (7.7% vs. 26.9%; *p* = 0.070). At 90 days, the TA group had significantly lower serum creatinine (105.6 µmol/L vs. 142.6 µmol/L; *p* = 0.015). Delayed graft function (15.4% vs. 11.5%; *p* = 1.000) and early postoperative renal function did not differ significantly.

**Conclusion:**

Tubeless anesthesia offers perioperative advantages and early graft function benefits in renal transplantation, reducing opioid/muscle relaxant requirements and accelerating recovery. Prospective large-scale studies are warranted to confirm its role in optimizing transplant outcomes.

**Trial registration:**

This study is a retrospective study.

## Introduction

Kidney transplantation is the optimal treatment for end-stage renal disease (ESRD), offering substantial improvements in patient survival and quality of life [1]. While advancements in surgical techniques have increased procedural success rates [2], anesthesia management continues to present significant challenges [3]. Endotracheal tube general anesthesia (ETT) remains the predominant approach for kidney transplantation globally [4] owing to its operational simplicity and safety profile. However, this method has notable drawbacks [5]. ETT elicits a pronounced stress response, which may lead to hemodynamic instability or cardiac complications [6–8] Furthermore, endotracheal intubation disrupts the respiratory mucosa’s natural barrier [7], and prolonged mechanical ventilation increases the risk of ventilator-associated pneumonia (VAP). ETT also necessitates high doses of anesthetic drugs, including muscle relaxants, sedatives, and opioids, drugs metabolized by the kidneys, potentially exacerbating toxicity to the transplanted organ [9]. Considering that it takes time for kidney transplant recipients to recover their renal function after surgery, postoperative recovery is usually long, and the awakening time is delayed. Frequent complications, such as sore throat and hoarseness [5], impair patient comfort and hinder rehabilitation.

The emerging paradigm of Tubeless surgery offers a transformative approach to surgical and perioperative management models [9–12] This strategy prioritizes physiological optimization and emphasizes precision medicine through individualized, physiologically driven management. Adapting this concept to kidney transplantation holds promise for refining anesthesia strategies, promoting patient early rehabilitation, and potentially improving long-term graft survival.

TA represents a pivotal innovation in this context. By replacing endotracheal intubation with a laryngeal mask, airway trauma can be prevented, and the hemodynamic stress response can be mitigated [13, 14]. In adult patients undergoing abdominal or head and cervicothoracic surgeries, ETT is associated with a higher incidence of postoperative complications such as hoarseness, cough, sore throat, and dysphagia [15, 16]. Regional nerve blocks, combined with titrated intravenous anesthesia, maintain adequate surgical conditions while reducing reliance on muscle relaxants and opioids [17]. In thoracic surgery, this anesthesia method helps maintain spontaneous breathing and rapid recovery after surgery and restores advanced cognitive functions as soon as possible, minimizing surgical trauma and anesthesia-related side effects, helping to shorten hospital stays and reduce medical costs [10, 18]. To date, only one study has briefly compared ventilation effectiveness and complications between ETT and TA in kidney transplantation [19]. No systematic analysis has been conducted regarding intraoperative anesthetic drug consumption, vital sign stability, or postoperative renal function. Considering the compromised renal function and immune status of transplant recipients, tubeless anesthesia may offer particular advantages in this setting.

In this study, we used a laryngeal mask airway instead of endotracheal intubation and combined intravenous anesthesia with regional blockade, which reduced the use of intraoperative anesthetic drugs (such as muscle relaxants and opioids), stabilized intraoperative hemodynamics, shortened the postoperative recovery time, effectively reduced the occurrence of respiratory complications, and improved the postoperative comfort of patients. This highlights the potential of the TA in optimizing transplant surgery and perioperative management.

## Materials and methods

### Case selection

We conducted a retrospective analysis of ESRD patients who underwent TA kidney transplantation at the First Affiliated Hospital of Guangzhou Medical University between July and December 2024. The donor kidneys were allocated via the China Organ Transplant Response System (COTRS). All the TA recipients met the predefined inclusion criteria during the preoperative anesthesia assessment. A total of 26 patients successfully underwent TA kidney transplantation. For comparative analysis, 26 contemporaneous ETT kidney transplant recipients were selected as controls. All the recipients who underwent TA kidney transplantation provided informed consent and signed the informed consent form.

### Inclusion criteria for tubeless anesthesia

(1) Body mass index (BMI) < 28 kg/m²; (2) absence of severe pulmonary dysfunction, confirmed by preoperative pulmonary function assessment and chest CT; (3) normal cardiac function (ejection fraction >50%) without significant arrhythmias (e.g., frequent atrial fibrillation or ventricular ectopy); (4) American Society of Anesthesiologists (ASA) classification ≤ III [20]; (5) ethically approved eligibility for kidney transplantation per review by the hospital’s human organ transplantation ethics committee; (6) appropriate donor kidneys were matched according to the distribution principles of the COTRS system [21].

### Anesthesia protocols

#### Tubeless anesthesia

##### Induction

 Sufentanil (0.1–0.2 µg/kg, intravenous), propofol (target-controlled infusion [TCI] 2–3 µg/mL). A double-lumen laryngeal mask was inserted at Bispectral Index (BIS) < 40.

##### Maintenance

Propofol (TCI 0.5–2.0 µg/mL), remifentanil (0.03–0.1 µg/kg/min), and dexmedetomidine (0.3–1.0 µg/kg/h) were used to maintain stable anesthesia.

##### Regional block

Bilateral transversus abdominis plane (TAP) blocks under ultrasound guidance with 15–20 mL 0.375% ropivacaine per side [22, 23]. 

##### Muscle relaxants

 Routinely use cisatracurium (initial bolus: 2–10 mg), titrated to surgical needs.

#### ETT anesthesia

Standard tracheal intubation with conventional general anesthesia was performed.

### Intraoperative monitoring

#### Hemodynamics

Continuous ECG, heart rate (HR), invasive arterial pressure (IBP), and central venous pressure (CVP). Real-time monitoring of circulatory status is needed to ensure circulatory stability and reduce the impact on the perfusion of the transplanted kidney.

#### Respiratory metrics

Pulse oximetry (SpO₂), end-tidal CO₂ (EtCO₂), tidal volume (VT), respiratory rate (RR), and Fraction of Inspiration O₂(FiO₂). Monitor the patient’s oxygenation status, assess ventilatory function to avoid hypoxemia, hypercapnia, or hypocapnia, and maintain physiologic breathing patterns.

#### Arterial blood gas (ABG)

Regular measurements of pH, PaO₂, PaCO₂, and lactate.

#### Anesthesia depth

The BIS monitors the depth of anesthesia, and the BIS is maintained at 40–60.

### Surgical technique

Kidney transplantation was performed extraperitoneally in the right iliac fossa with end-to-side arterial/venous anastomoses and Lich‒Gregoir ureteroneocystostomy. Intraoperative complications (such as intraoperative hemorrhage, hypotensive state, hypoxemia, hypercapnia, and cardiac arrest), operation time, and postoperative anesthesia recovery time were recorded for both groups.

### Postoperative assessments

(1) Pain assessment Visual Analog Scale (VAS) [24] is used to quantify the degree of postoperative pain, guide the use of analgesic drugs and optimize pain management.

(2) Airway complications: airway-related complications such as sore throat, hoarseness, or hemoptysis in postoperative patients.

 (3) Graft function and Non-airway complications: Record Serum creatinine (Scr) and estimated glomerular filtration rate (eGFR, Cockcroft-Gault) [25], and non-airway complication (e.g. delayed graft function [DGF], acute rejection, infection).

### Follow-up

Graft function (Scr, eGFR) and complications (e.g., acute rejection, infections, cardiovascular events) were regularly monitored for 90 days of follow-up.

### Statistical analysis

SPSS 22.0 was used for analyses. Normally distributed data (mean ± SD) were compared via *t*- tests; non-normally distributed data (median [IQR]) via Mann-Whitney U tests. Categorical variables (n [%]) were analyzed with χ² or Fisher’s exact tests. Two-sided *p* < 0.05 defined significance.

## Results

### Baseline characteristics

Groups were well matched (shown in Table 1). The mean BMI of the TA group was less than 24, the preoperative cardiac function was good, and the left ventricular ejection fraction (LVEF) was greater than 50% of all patients. In the donor category, there were 2 living donors in the TA group, 1 living donor in the ETT group, and the rest were deceased donors. There were 15 deceased donors in both groups, all of whom donated DBDs (Donors of Brain Death). *In both* groups, donors and recipients were fully matched for both ABO and Rh blood types. All patients who tested positive for panel reactive antibodies (PRA) were weakly positive, and during HLA matching, sensitized epitopes were intentionally avoided. Consequently, none of the patients required preoperative desensitization therapy. The immune induction strategy of both groups was antithymocyte globulin (ATG) and methylprednisolone (MP) or basiliximab and MP. The postoperative immune maintenance strategy for both groups was the classic calcineurin inhibitor + mycophenolate mofetil + prednisolone (CNI + MMF + PRED) triple regimen [26].

**Table 1:** Baseline characteristics in both groups

	TA(*N* = 26)	ETT(*N* = 26)	*P*
Gender (N)			*0.061*
Female	10(38.5%)	4(15.4%)	
Male	16(61.5%)	22(84.6%)	
Age (Years, M ± SD)	42.3 ± 12.3	41.2 ± 9.9	*0.738*
BMI(Kg/m^2^, M ± SD)	22.1 ± 3.6	23.0 ± 3.5	*0.475*
EF value(%, M ± SD)	67.5 ± 6.5	65.2 ± 6.4	*0.212*
Blood type (N)			*0.394*
A	6(23.1%)	11(42.3%)	
B	5(19.2%)	3(11.5%)	
O	10(38.5%)	6(23.1%)	
AB	5(19.2%)	6(23.1%)	
Donor Type (N)			*1.000*
Living donor	2(11.8%)	1(6.3%)	
Deceased donor	15(88.2%)	15(93.8%)	
Donor gender (N)			*0.915*
Female	4(23.5%)	5(31.3%)	
Male	13(76.5%)	11(68.8%)	
Donor Age (Years, M ± SD)	41.2 ± 14.1	40.9 ± 15.1	*0.963*
Donor cause of death(N)			*0.417*
Cerebrovascular accident	8(53.3%)	4(26.7%)	
Brain trauma	6(40.0%)	9(60.0%)	
Hypoxic-ischemic encephalopathy	1(6.7%)	2(13.3%)	
PRA(N)			*0.193*
Negative	21(80.8%)	25(96.2%)	
Positive	5(19.2%)	1(3.8%)	
HLA mismatch (N, M ± SD)	4.0 ± 1.4	4.0 ± 1.1	*0.874*
Immune induction strategy (N)			*0.165*
ATG + MP	16(61.5%)	11(42.3%)	
Basiliximab + MP	10(38.5%)	15(57.7%)	
Immune maintenance strategy (N)			*1.000*
Tac + MMF/MPS + Pred	23(88.5%)	22(84.6%)	
CsA + MMF/MPS + Pred	3(11.5%)	4(15.4%)	
Warm ischemia time (min, M ± SD)	0.4 ± 1.5	1.1 ± 3.1	*0.567*
Cold ischemia time (hour, M ± SD)	6.3 ± 5.1	6.9 ± 5.7	*0.389*

(This table is referenced in line 152 of the text)

*BMI Body Mass Index, *EF value* Ejection Fraction valu, *PRA* Panel reactive antibodie, *ATG* Anti-thymocyte Globulin, *MP* Methylprednisolon, *Tac* Tacrolimus,*MMF/MPS* Mycophenolate Mofetil/Mycophenolic Acid Sodium, *CsA* Cyclosporine A, *Pred* Prednisone

### Intraoperative outcomes

Kidney transplantation was successfully completed in both groups, and there were no cases of intraoperative conversion to endotracheal intubation in the TA group. TA reduced cisatracurium use by 51.7% (12.6 ± 10.3 vs. 26.1 ± 6.8 mg, *p* < 0.05) and sufentanil use by 22.3% (22.3 ± 11.2 vs. 28.7 ± 8.7 µg, *p* < 0.05). However, there were no significant differences in the operative time (211.5 ± 45.7 min vs. 200.8 ± 35.5 min, *p* > 0.05) or anesthesia time (296.6 ± 57.4 min vs. 292.7 ± 48.8 min, *p* > 0.05).

Intraoperative monitoring revealed that the vital signs of the patients in both groups were generally stable, and no serious complications, such as arrhythmia, hypoxemia, or hypercapnia, occurred. Vasopressor requirements tended to be lower in TA group (14 vs. 19 patients; Table 2), and only 1 patient needed to use two vasoactive drugs in combination (4 patients in the ETT group). The dosage of dopamine, the most commonly used drug, tended to decrease in the TA group, but *p* > 0.05 (Table 2; Fig. 1C/D). The incidence of intraoperative complications in both groups was 1 case (Table 2). In the TA group, one patient required emergency intervention due to graft subcapsular hemorrhage. And one patient in the TA group versus two in the ETT group were transfused for low hemoglobin on intraoperative blood gas analysis. Each received < 2 units. Transfusion rates did not differ significantly (*p* > 0.05). The patient’s vital signs were stable after surgery, but DGF occurred. In the ETT group, one patient experienced persistent hypotension (the lowest systolic blood pressure was 60 mmHg, which lasted for 15 min), and DGF also occurred postoperation. The postoperative anesthesia recovery time in the TA group was significantly shorter than that in the ETT group (18.5 ± 10.8 min vs. 34.4 ± 14.7 min, *p* < 0.05) (Table 2; Fig. 1E), but there was no significant difference in the postoperative pain score (VAS score) (Table 2; Fig. 1F).

**Table 2:** Intraoperative data and postoperative recovery data for both groups

	TA (*N* = 26)	ETT (*N* = 26)	*P*
Operative Time (min, M ± SD)	211.5 ± 45.7	200.8 ± 35.5	*0.949*
Anesthesia Time (min, M ± SD)	296.6 ± 57.4	292.7 ± 48.8	*0.628*
Blood Loss (mL, M ± SD)	109.6 ± 133.7	72.7 ± 34.1	*0.529*
Urine Output (mL, M ± SD)	236.5 ± 313.8	224.1 ± 229.7	*0.507*
Anesthetic Drug Usage			
Muscle Relaxant			
Cisatracurium (mg, M ± SD)	12.6 ± 10.3	26.1 ± 6.8	***0.000***
Analgesics			
Remifentanil (mg, M ± SD)	1460.4 ± 704.8	1365.6 ± 572.5	*0.647*
Sufentanil (mg, M ± SD)	22.3 ± 11.2	28.7 ± 8.7	***0.007***
Sedatives			
Midazolam (mg, M ± SD)	3.1 ± 1.3	2.8 ± 0.6	*1.000*
Dexmedetomidine (mg, M ± SD)	87.2 ± 45.8	80.9 ± 39.8	*0.597*
Types of Vasoactive Drugs(N)			*0.211*
None	12(46.2%)	7(26.9%)	
One Type	13(50%)	15(57.7%)	
Two Types	1(3.8%)	4(15.4%)	
Dosage of Vasoactive Drugs			
Dopamine (µg, M ± SD)	34347.7 ± 18941.4	48785.5 ± 25196.9	*0.093*
#Intraoperative Complications(N)	1(3.9%)	1(3.9%)	
Postoperative Anesthesia Recovery Time (min, M ± SD)	18.5 ± 10.8	34.4 ± 14.7	***0.000***
Postoperative Pain Score (VAS Score)	3.3 ± 1.0	2.8 ± 0.9	*0.066*
DGF	4(15.4%)	3(11.5%)	*1.000*
Postoperative Complications(N)			
Airway Complications	2(7.7%)	7(26.9%)	*0.143*
Non airway Complications	7(26.9%)	10(38.5%)	*0.375*
Renal function			
Serum Creation(umol/L, M ± SD)			
Preoperation	860.8 ± 299.5	1091.5 ± 283.6	***0.008***
POD7	267.8 ± 203.4	245.0 ± 240.8	*0.516*
POD30	132.6 ± 59.4	158.4 ± 90.5	*0.194*
POD90	105.6 ± 24.3	142.6 ± 44.4	***0.015***
Estimated Glomerular Filtration Rat(Cockcroft-Gault, ml/min/1.73 m², M ± SD)			
Preoperation	6.4 ± 2.1	5.1 ± 1.9	***0.014***
POD7	43.0 ± 35.2	46.0 ± 25.8	*0.462*
POD30	59.5 ± 25.2	53.5 ± 22.2	*0.378*
POD90	66.6 ± 14.6	55.6 ± 21.5	*0.056*
Rejection (within three months)			*0.235*
No rejection	26(100%)	23(88.5%)	
Acute rejection	0(0)	3(11.5%)	
Blood transfusion			*1.000*
None	25(96.2%)	24(92.3%)	
Yes	1(3.8%)	2(7.7%)	

(This table is referenced in line 174–199/229 of the text)

* *VAS Score* Visual Analogue Scale Score, *DGF* Delayed Graft Function, *POD* Post Operative Day

# Intraoperative Complications: TA group: Subcapsular hemorrhage in the transplanted kidney; ETT group: Intraoperative hypotension

**Fig. 1:**
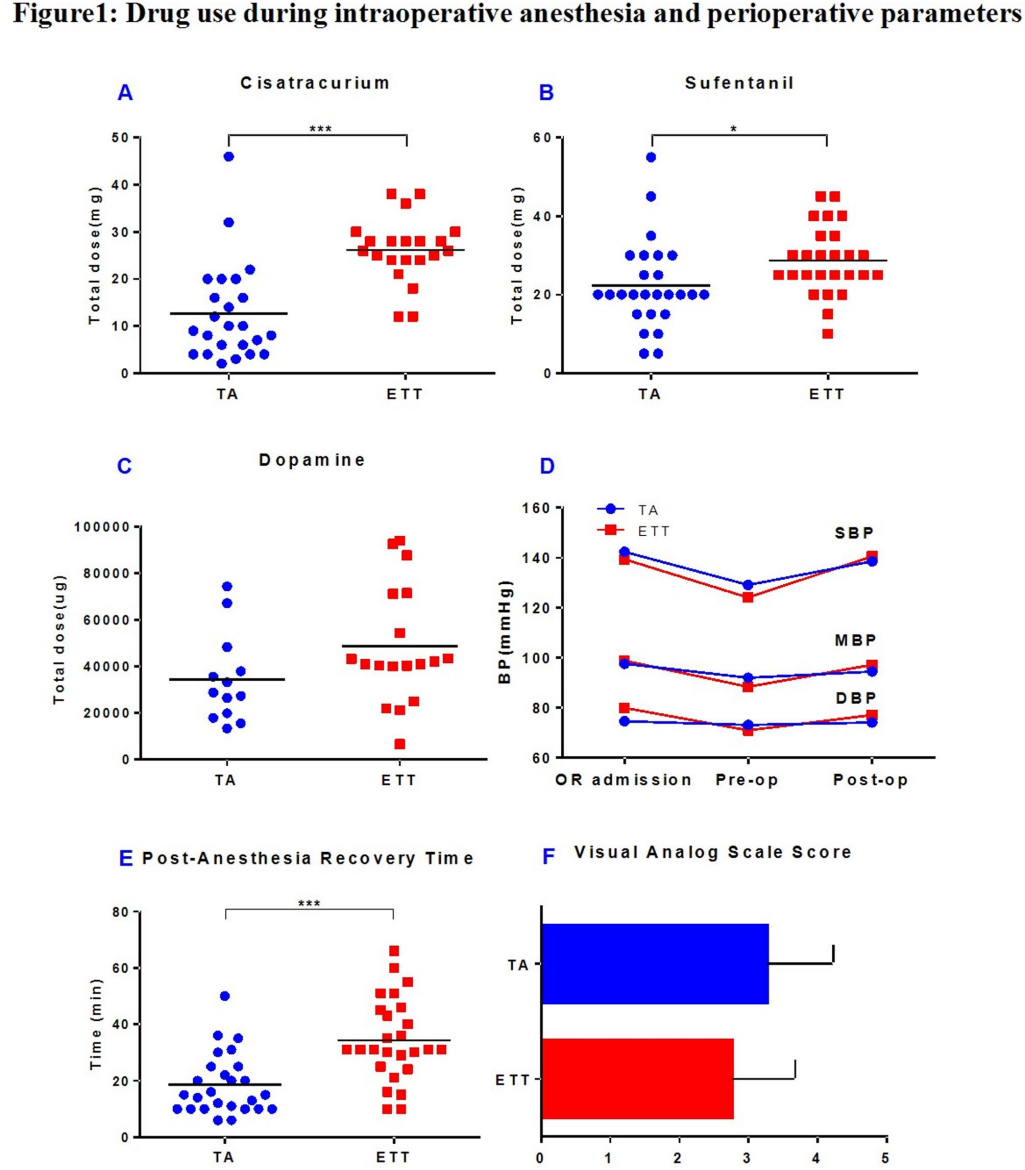
Drug use during intraoperative anesthesia and perioperative parameters.**A** The usage of intraoperative muscle relaxant drugs (cisatracurium) in the TA group decreased significantly (12.63 ± 10.34 mg vs. 26.14 ± 6.84 mg, *p* < 0.05). **B** The TA group also showed a significant reduction in the intraoperative dosage of the potent opioid analgesic sufentanil (22.31 ± 11.16 mg vs. 28.65 ± 8.67 mg, *p* < 0.05). **C and D** Both groups maintained stable blood pressure during the operation, the blood pressure fluctuations in the TA group were more straight and intraoperative dopamine dosage tended to decrease, but the difference was not statistically significant (*P*>0.05). **E** The postoperative anesthesia recovery time in the TA group was significantly shorter than ETT group (18.54 ± 10.75 min vs. 34.35 ± 14.69 min, *p* < 0.05). **F **There was no statistical difference in postoperative pain scores(VAS score) between the two groups(*P*>0.05)

### Postoperative outcomes

**Fig. 2:**
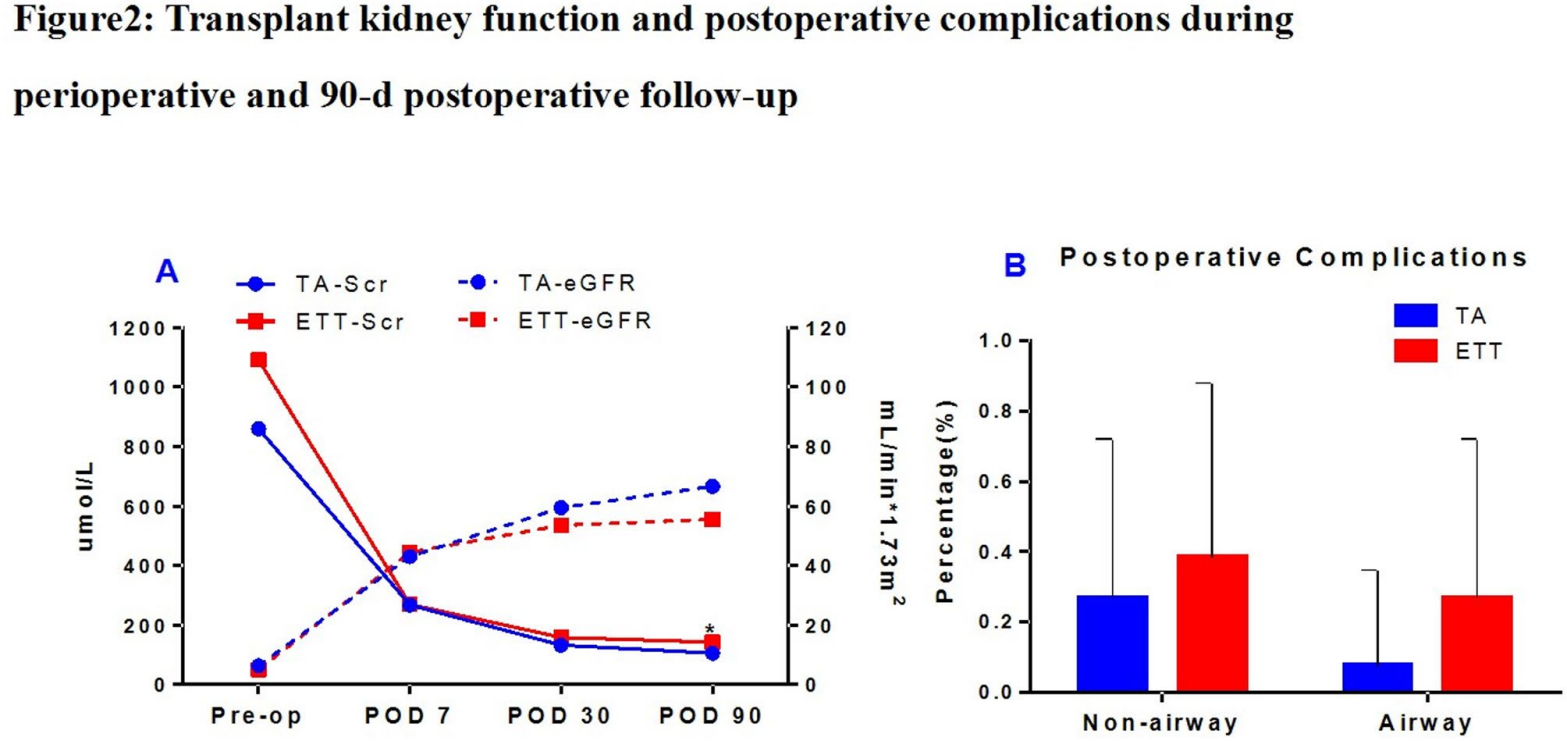
Transplant kidney function and postoperative complications during perioperative and 90-d postoperative follow-up.**A** The renal function of the transplanted kidneys (serum creatinine and estimated glomerular filtration rate [eGFR]) in both groups showed a gradual improvement trend after surgery, except for the blood creatinine value at pod90, there was no significant difference between the two groups for other parameters. **B** The postoperative complication rates during the perioperative period and follow-up were similar in both groups. The incidence of perioperative airway-related complications in the TA group was 2 cases (7.7%), which was lower than 7 cases (26.9%) in the ETT group. The incidence of postoperative non-airway complications was 7 cases (26.9%) and 10 cases (38.5%) respectively. No statistical difference was observed in both complications (*p* > 0.05)

DGF incidence was similar between the two groups (TA: 15.4% vs. ETT: 11.5%, *p* > 0.05) (Table 2), and recovery of renal function was observed in all DGF recipients. Postoperative follow-up at 7, 30, and 90 days revealed a gradual improvement in renal function, as indicated by serum creatinine levels and eGFRs in both groups. However, no significant differences were observed between groups except for serum creatine at 90 days (Table 2; Fig. 2A). At 90 days, serum creatine concentration was 105.6 ± 24.3 µmol/L in the TA group, which was significantly lower than that in the ETT group (142.6 ± 44.4 µmol/L, *p* < 0.05). The mean eGFR of the TA group at 90 days was also 11.04 mL/min higher than that of the ETT group (*p* > 0.05). No acute rejection occurred in the TA group versus 3 cases in the ETT group within 3 months and the difference was not statistically significant (*p* > 0.05). In addition, the overall perioperative and follow-up complication rates were similar in both groups (Table 2). The incidence of perioperative airway-related complications in the TA group was 2 (7.7%), which was lower than 7 (26.9%) that in the ETT group. The incidence rates of postoperative non-airway complications were 7 (26.9%) and 10 (38.5%), respectively, with no significant difference (*p* > 0.05).

## Discussion

By minimizing airway trauma, reducing perioperative stress, and accelerating recovery, TA aligns with enhanced recovery after surgery (ERAS) protocols, offering tangible improvements in postoperative comfort and quality of life [18, 26]. The integration of the concept of tubeless surgery into renal transplantation represents both a technical innovation and a paradigm shift toward patient-centered treatment. Moreover, these advantages may have a positive impact on the long-term function of the transplanted kidney and the quality of life of patients, providing a new direction for the development of renal transplant surgery. Guided by the tubeless surgical concept, TA represents the initial step undertaken by our center toward achieving tubeless kidney transplantation (TKT), with preliminary clinical results demonstrating promising advantages. The advantages are multifaceted, encompassing both intraoperative and postoperative benefits.

Cisatracurium, a widely utilized muscle relaxant in clinical practice, results in reduced clearance in patients with ESRD [11], consequently increasing the risk of postoperative residual neuromuscular blockade [27–29] and prolonging anesthesia recovery time. Compared with the ETT group, the implementation of the tubeless anesthesia protocol achieved a 51.7% reduction in the average intraoperative cisatracurium dosage while maintaining satisfactory intraoperative neuromuscular blockade. Notably, no intergroup difference was observed in surgical duration. Postoperatively, the TA group demonstrated a 46.0% reduction in average anesthesia recovery time, accompanied by accelerated restoration of consciousness and orientation. Fenta nil, another commonly employed anesthetic agent, belongs to the class of potent opioid analgesics. Although primarily metabolized by the liver, cumulative dosing carries inherent risks of adverse effects, including respiratory depression, bradycardia, and hypotension [30, 31], which may compromise graft perfusion. TA protocol implementation resulted in a 22.3% reduction in intraoperative sufentanil consumption, enhanced hemodynamic stability [32], and reduced reliance on vasoactive agents (shown in Table 2). Notably, postoperative pain assessment via the VAS demonstrated no statistically significant differences in pain scores between the groups.

During surgery, patients in the TA group were managed with a laryngeal mask airway (LMA) instead of endotracheal intubation, avoiding invasive endotracheal injury and hemodynamic fluctuations associated with intubation stimuli. Compared with the ETT group, the TA group demonstrated effective ventilation, more stable circulatory parameters [33], and a reduced need for vasoactive agents, with no serious intraoperative complications observed. Postoperatively, patients reported improved comfort, with fewer endotracheal intubation-related complications, such as sore throat and hoarseness. These benefits facilitate early oral intake and ambulation, effectively accelerating gastrointestinal recovery and preventing deep vein thrombosis, thereby promoting overall postoperative recovery.

Notably, at the postoperative follow-up, the blood creatinine level in the TA group was significantly better than that in the ETT group at 90 days, indicating a potential advantage in improving the long-term eGFR. The TA technique may have a beneficial effect on the long-term prognosis of transplanted kidneys by optimizing perioperative management. The implementation of TA eliminates the use of inhalation anesthetics, substantially reduces the dosages of muscle relaxants and analgesics, and maintains greater intraoperative hemodynamic stability. Consequently, it minimizes the nephrotoxic effects of anesthetic agents on the transplanted kidney [34], resulting in significantly enhanced renal function at 90 days postoperation. However, as this study is retrospective, further validation through multicenter randomized controlled trials is warranted.

A series of issues are also faced during the execution of this study that need to be continuously refined and improved. First, the inclusion and exclusion criteria for TA kidney transplantation need to be optimized. The current inclusion criteria were adapted from thoracic surgery^[15]^, but renal transplantation is an abdominal surgery, which generally does not directly affect the function of thoracic organs. Renal transplantation patients who have received renal replacement therapy for a long period before surgery often have comorbidities such as hypertension, coronary artery disease, diabetes mellitus, and other underlying diseases. Therefore, successful application of the TA requires a multidisciplinary collaborative mechanism. Second, the effectiveness of TA depends on anesthetic expertise and team coordination, given that technical challenges such as respiratory depression, difficult airway management, and precise control of anesthetic depth all require close surgeon‒anesthesiologist collaboration and a high level of professional competence. Third, the safe implementation of TAs relies on the availability of advanced monitoring systems and emergency equipment to provide the necessary technical support.

## Conclusions

The successful application of the TA technique in renal transplantation based on the tubeless surgery concept not only accelerates patient recovery and saves medical resources but also may have a positive effect on the long-term prognosis of renal transplant recipients. Moreover, the implementation of TA technology is a systems project that requires the establishment of standardized operating procedures and a multidisciplinary consensus mechanism at the hospital level to ensure the stability of the technology and the safety of the patients. In the future, preserving spontaneous breathing via the TA may reduce the degree of lung injury caused by mechanical ventilation [35] and further reduce the dosage of muscle relaxants and anesthetic drugs to achieve greater benefits for kidney transplant patients. Therefore, TA technology still needs to be continuously optimized and improved to promote its wide application in renal transplantation.

This study is a single-center, retrospective case‒control study with a short follow-up period and a lack of blinding, which may limit the generalizability of the findings. Therefore, the results should be interpreted with caution. Further large-scale, multicenter, prospective randomized controlled trials with longer follow-up periods are needed to validate these findings.

## Data Availability

All data in this article are from the clinical information management system of the Organ Transplantation Department of the First Affiliated Hospital of Guangzhou Medical University。 All data generated or analyzed in this study are available by contacting the corresponding author.

## Abbreviations

TA: Tubeless anesthesia
ETT: Endotracheal tube
ESRD: End-stage renal disease
VAP: Ventilator-associated pneumonia
COTRS: China Organ aTransplant Response System
BMI: Body mass index
ASA: American Society of Anesthesiologists
TCI: Target-controlled infusion
BIS: Bispectral Index
TAP: Transversus abdominis plane
HR: Heart rate
IBP: Invasive arterial pressure
CVP: Central venous pressure
SpO₂: Pulse oximetry
EtCO₂: End-tidal CO₂
VT: Tidal volume
RR: Respiratory rate
FiO₂: Fraction of Inspiration O₂
ABG: Arterial Blood Gas
VAS: Visual Analog Scale
Scr: Serum creatinine
eGFR: Estimated glomerular filtration rate
DGF: Delayed graft function
LVEF: Left Ventricular Ejection Fraction
ATG: Antithymocyte globulin
MP: Methylprednisolone
CNI: Calcineurin inhibitors
MMF: Mycophenolate mofetil
PRED: Prednisolone
ERAS: Enhanced Recovery After Surgery
TKT: Tubeless Kidney Transplantation
LMA: Laryngeal mask airway

## References

[1] Nieto T, Inston N, Cockwell P. Renal transplantation in adults. BMJ (Clinical research ed). 2016;355:i6158. 27903494 10.1136/bmj.i6158

[2] Lentine KL, Smith JM, Miller JM, Bradbrook K, Larkin L, Weiss S, et al. OPTN/SRTR 2021 annual data report: kidney. Am J Transplant. 2023;23(2 Suppl 1):S21–120. 37132350 10.1016/j.ajt.2023.02.004 PMC9970360

[3] Kim H, Jung H. Considerations regarding anesthesia for renal transplantation. Anesth Pain Med. 2024;19(1):5–11. 10.17085/apm.23153 PMC10847005 38311350

[4] Nawabi A, Sullivan P, De Ruyter M, Pichoff A, King CD, Nawabi P. Surgical approach for kidney transplantation under spinal anesthesia. J Surg Case Rep. 2020 Dec 31;2020(12):rjaa538. 10.1093/jscr/rjaa538 PMC7778361 33425319

[5] Dong W, Zhang W, Er J, Liu J, Han J. Comparison of laryngeal mask airway and endotracheal tube in general anesthesia in children. Exp Ther Med. 2023;26(6):554. 37941592 10.3892/etm.2023.12253 PMC10628640

[6] Shribman AJ, Smith G, Achola KJ. Cardiovascular and catecholamine responses to laryngoscopy with and wi thout tracheal intubation. Br J Anaesth. 1987;59(3):295–9. 3828177 10.1093/bja/59.3.295

[7] Ozdemir E, Kavakli O. Risk factors for oral mucosal pressure injury associated with endotrac heal tubes in intensive care unit patients: a single-centre longitudin al study with brief follow-up. Nurs Crit Care. 2025;30(2):e70009. 40068960 10.1111/nicc.70009

[8] Russotto V, Myatra SN, Laffey JG, Tassistro E, Antolini L, Bauer P, et al. Intubation practices and adverse peri-intubation events in critically ill patients from 29 countries. JAMA. 2021;325(12):1164–72. 33755076 10.1001/jama.2021.1727 PMC7988368

[9] Jaszczuk S, Natarajan S, Papalois V. Anaesthetic approach to enhanced recovery after surgery for kidney Tra nsplantation: a narrative review. J Clin Med. 2022;11(12):3435. 35743505 10.3390/jcm11123435 PMC9225521

[10] He J, Liang H, Wang W, Akopov A, Aiolfi A, Ang K-L, et al. Tubeless video-assisted thoracic surgery for pulmonary ground-glass no dules: expert consensus and protocol (Guangzhou). Transl Lung Cancer Res. 2021;10(8):3503–19. 34584853 10.21037/tlcr-21-663 PMC8435391

[11] Li J, Liu J, Hamblin L, Liu H, Liang L, Dong Q, et al. Simple to simplest: the tubeless technique. J Thorac Dis. 2017 Feb;9(2):222–4. 10.21037/jtd.2017.02.55 PMC5334079 28275463

[12] Solli P, Brandolini J, Bertolaccini L. Tubeless thoracic surgery: ready for prime time? J Thorac Dis. 2019;11(3):652–6. 31019751 10.21037/jtd.2019.03.01 PMC6462670

[13] Perelló-Cerdà L, Fàbregas N, López AM, Rios J, Tercero J, Carrero E, et al. Proseal laryngeal mask airway attenuates systemic and cerebral hemodyn amic response during awakening of neurosurgical patients: a randomized clinical trial. J Neurosurg Anesthesiol. 2015;27(3):194–202. 25121397 10.1097/ANA.0000000000000108

[14] Jarineshin H, Kashani S, Vatankhah M, Abdulahzade Baghaee A, Sattari S, Fekrat F. Better hemodynamic profile of laryngeal mask airway insertion compared to laryngoscopy and tracheal intubation. Iran Red Crescent Med J. 2015;17(8):e28615. 26430529 10.5812/ircmj.28615 PMC4587401

[15] He J, Liu J, Zhu C, Dai T, Cai K, Zhang Z et al. Expert consensus on tubeless video-assisted thoracoscopic surgery (Gua ngzhou). J Thorac Dis. 11(10):4101–8. 10.21037/jtd.2019.10.04 PMC6837991 31737292

[16] Xu R, Lian Y, Li WX. Airway complications during and after general anesthesia: a comparison, systematic review and meta-analysis of using flexible laryngeal mask airways and endotracheal tubes. PLoS ONE. 2016;11(7):e0158137. 27414807 10.1371/journal.pone.0158137 PMC4944923

[17] Wiederhold BD, Garmon EH, Peterson E, Stevens JB, O’Rourke MC. Nerve block anesthesia. StatPearls: StatPearls Publishing; 2023. 28613761

[18] de Carvalho CC, Kapsokalyvas I, El-Boghdadly K. Second-generation supraglottic airway devices versus endotracheal intu bation in adults undergoing abdominopelvic surgery: a systematic revie w and meta-analysis. Anesth Analg. 2025;140(2):265–75. 39466638 10.1213/ANE.0000000000006951

[19] Zhou Y, Liang H, Xu K, Yang C, Liang L, Dong Q, et al. The strategy of non-intubated spontaneous ventilation anesthesia for u pper tracheal surgery: a retrospective case series study. Transl Lung Cancer Res. 2022;11(5):880–9. 35693283 10.21037/tlcr-22-302 PMC9186174

[20] Horvath B, Kloesel B, Todd MM, Cole DJ, Prielipp RC. The evolution, current value, and future of the American society of an esthesiologists physical status classification system. Anesthesiology. 2021;135(5):904–19. 34491303 10.1097/ALN.0000000000003947

[21] Huang J, Wang H, Fan ST, Zhao B, Zhang Z, Hao L, et al. The national program for deceased organ donation in China. Transplantation. 2013;96(1):5–9. 23743728 10.1097/TP.0b013e3182985491

[22] Park S, Kim GS, Choi DH, Ko JS, Park JB, Son YH, et al. Comparison of pulmonary gas exchange during kidney transplantation: se cond-generation laryngeal mask airway vs endotracheal tube. Transplant Proc. 2020;52(6):1695–9. 32336651 10.1016/j.transproceed.2019.12.057

[23] Tran DQ, Bravo D, Leurcharusmee P, Neal JM. Transversus abdominis plane block: a narrative review. Anesthesiology. 2019;131(5):1166–90. 31283738 10.1097/ALN.0000000000002842

[24] He S, Renne A, Argandykov D, Convissar D, Lee J. Comparison of an emoji-based visual analog scale with a numeric rating scale for pain assessment. JAMA. 2022;328(2):208–9. 35819433 10.1001/jama.2022.7489 PMC9277495

[25] Michels WM, Grootendorst DC, Verduijn M, Elliott EG, Dekker FW, Krediet RT. Performance of the Cockcroft-Gault, MDRD, and new CKD-EPI formulas in relation to GFR, age, and body size. Clin J Am Soc Nephrol. 2010;5(6):1003–9. 20299365 10.2215/CJN.06870909 PMC2879308

[26] Ekberg H, Tedesco-Silva H, Demirbas A, Vítko S, Nashan B, Gürkan A, et al. Reduced exposure to calcineurin inhibitors in renal transplantation. N Engl J Med. 2007 Dec 20;357(25):2562–75. 10.1056/NEJMoa067411 18094377

[27] Della Rocca G, Pompei L, Coccia C, Costa MG, Cecchini V, Vilardi V, et al. Atracurium, cisatracurium, vecuronium and rocuronium in patients with renal failure. Minerva Anestesiol. 2003;69(7–8):605–11, 612, 5. 14564242

[28] Robertson EN, Driessen JJ, Booij LHDJ. Pharmacokinetics and pharmacodynamics of rocuronium in patients with a Nd without renal failure. Eur J Anaesthesiol. 2005;22(1):4–10. 15816565 10.1017/s0265021505000025

[29] Craig RG, Hunter JM. Neuromuscular blocking drugs and their antagonists in patients with or Gan disease. Anaesth. 64 Suppl 1:55–65. 10.1111/j.1365-2044.2008.05871.x 19222432

[30] Khuenl-Brady KS, Pomaroli A, Pühringer F, Mitterschiffthaler G, Koller J. The use of Rocuronium (ORG 9426) in patients with chronic renal Failur e. Anaesthesia. 48(10):873–5. 10.1111/j.1365-2044.1993.tb07417.x 8238829

[31] Pattinson KTS. Opioids and the control of respiration. Br J Anaesth. 2008;100(6):747–58. 18456641 10.1093/bja/aen094

[32] Scott JC, Cooke JE, Stanski DR. Electroencephalographic quantitation of opioid effect: comparative pha rmacodynamics of Fentanyl and sufentanil. Anesthesiology. 1991;74(1):34–42. 1670913 10.1097/00000542-199101000-00007

[33] Wei C-F, Chung Y-T. Laryngeal mask airway facilitates a safe and smooth emergence from Ane sthesia in patients undergoing craniotomy: a prospective randomized Co ntrolled study. BMC Anesthesiol. 2023;23(1):29. 36650435 10.1186/s12871-023-01972-x PMC9843947

[34] Hauquiert B, Gonze A, Gennart T, Perriens E, Blackman S, De Lissnyder N, et al. Nephrotoxicity and modern volatile anesthetics: a narrative review. Toxics. 2025. 10.3390/toxics13060514. 40559988 10.3390/toxics13060514 PMC12197702

[35] Silva PL, Ball L, Rocco PRM, Pelosi P. Physiological and Pathophysiological Consequences of Mechanical Ventilation. Semin Respir Crit Care Med. 2022 Jun;43(3):321–34. 10.1055/s-0042-1744447 35439832

